# Dorsalis Pedis Free Flap: The Salvage Option following Failure of the Radial Forearm Flap in Total Lower Lip Reconstruction

**DOI:** 10.1155/2014/458286

**Published:** 2014-04-02

**Authors:** Theodoros Stathas, Georgios Tsinias, Dimitra Tsiliboti, Aris Tsiros, Nicholas Mastronikolis, Panos Goumas

**Affiliations:** ^1^Department of Otolaryngology, Head and Neck Surgery, University Hospital of Patras, 26504 Patras, Greece; ^2^Department of Plastic and Reconstructive Surgery, “Agios Andreas” General Hospital of Patras, 26335 Patras, Greece

## Abstract

Reconstruction after resection of large tumors of the lower lip requires the use of free flaps in order to restore the shape and the function of the lip, with the free radial forearm flap being the most popular. In this study we describe our experience in using the dorsalis pedis free flap as a salvage option in reconstruction of total lower lip defect in a patient with an extended lower lip carcinoma after failure of the radial forearm free flap, that was initially used. The flap was integrated excellently and on the followup the patient was free of disease and fully satisfied with the aesthetic and functional result.

## 1. Introduction

Treatment of choice for lower lip cancer is wide full thickness excision and reconstruction of the remaining defect. Extended lower lip defects resulting after excision of large tumours (involving >2/3 of the lip) represent a very difficult reconstructive problem and include the use of locoregional flaps or free flaps. The aim of reconstruction is closure of the defect and restoration of lip function. Regional flaps like bilateral advancement flaps, Karapandzic flap, and Gilles fan flap are not effective because they result in microstomia and create extended facial scarring and dysmorphia [1–3].

Total lower lip excision defects can only be satisfactorily closed with the use of composite free flaps [4–9]. The ideal free flap should be thin and pliable in order to be shaped to reconstruct the lower lip and provide sensitivity and support in order to avoid drooling. The free flap that gathers all these properties is the free radial forearm flap, and thus it is the most commonly used free flap in the case of reconstruction of the lower lip. Flap necrosis is an unfortunate event that has to be confronted with the use of another flap. The substitute flap can be the dorsalis pedis free flap, since it resembles in its characteristics the radial forearm flap. We present our experience with the use of the dorsalis pedis flap as an alternative composite fasciocutaneous tendon flap in total lower lip reconstruction.

## 2. Materials and Methods

**Figure 1:**
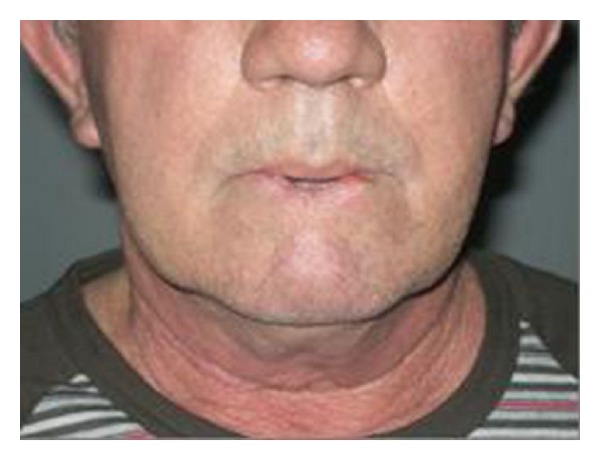
Extended SCC of the lower lip.

**Figure 2:**
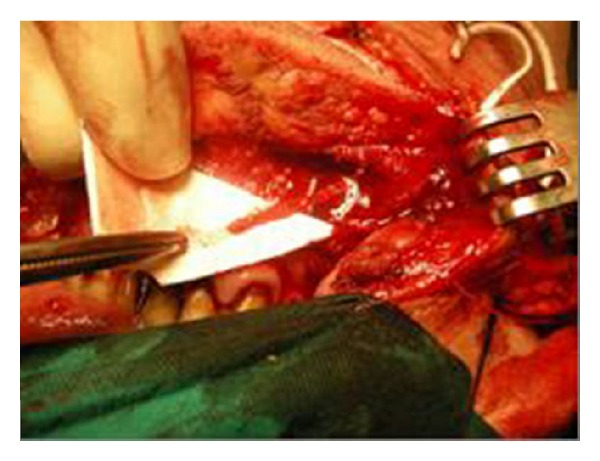
Excision of the lower lip with preservation of the mental nerve.

**Figure 3:**
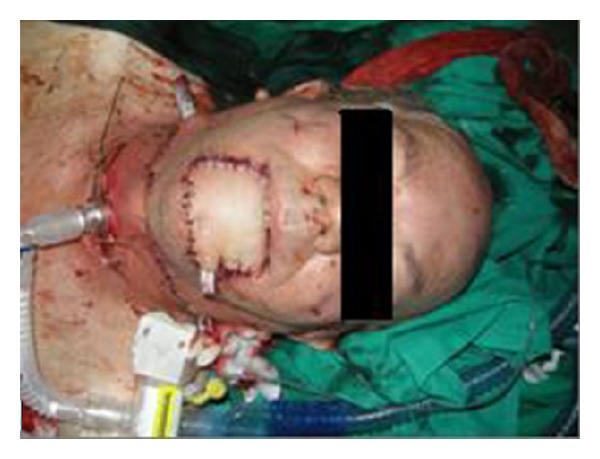
Reconstruction with the free composite radial forearm-palmaris longus flap.

**Figure 4:**
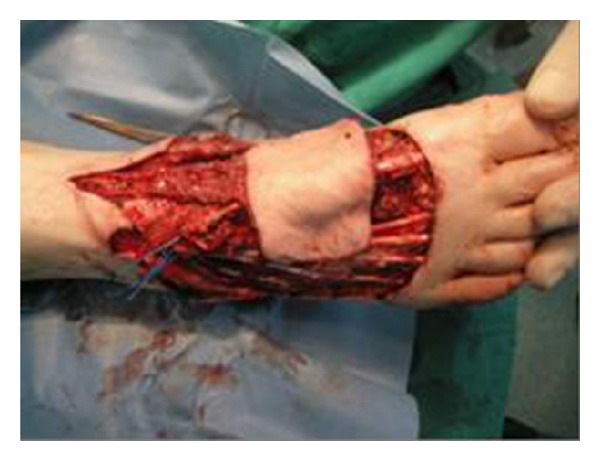
Harvesting of the free composite dorsalis pedis-extensor hallucis brevis flap.

**Figure 5:**
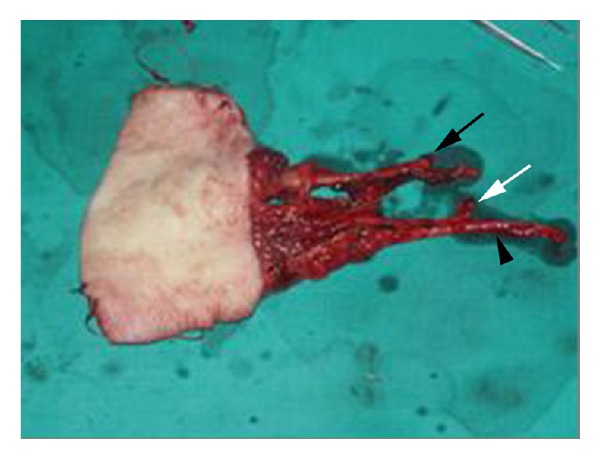
The free composite dorsalis pedis-extensor hallucis brevis flap (black arrow: dorsalis pedis artery and vein, white arrow: extensor hallucis brevis tendon, and arrowhead: saphenous vein).

**Figure 6:**
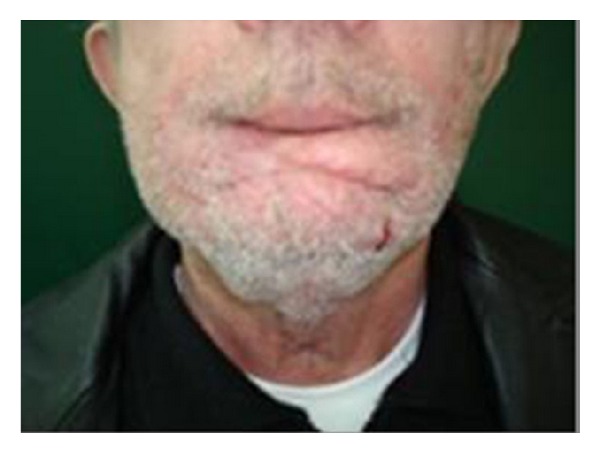
Final result 1 year post-op.

We present the case of a 65-year-old male patient with extended lower lip cancer involving more than 2/3 of the lower lip (Figure 1). Biopsy showed a high grade squamous cell carcinoma and CT scan of the neck revealed no lymph node metastases. The patient underwent total lower lip excision and bilateral supraomohyoid selective neck dissection (Figure 2). A composite radial forearm-palmaris longus free flap was harvested from the right arm according to the size of the oral mucosa and external face defect. The flap was folded around the tendon which was transected 5 cm from both ends of the flap in order to provide sufficient length for the anchorage. The flap was properly inserted and sutured to restore the defect and microanastomoses were performed between radial and facial artery and vein. The lateral cutaneous radial nerve which was harvested with the flap was sutured to mental nerve in order to restore lower lip sensation. Both ends of the tendon were passed subcutaneously through the cheek bilaterally to be sutured to the remaining orbicularis oris muscle and the periosteum of the malar eminence. The tension of the tendon suturing was adjusted to provide good oral competence so that satisfactory closure of the mouth could be passively achieved (Figure 3). The donor site was reconstructed with split-thickness skin graft. The free flap failed to survive due to late vein thrombosis on the 5th post-op day. The left radial forearm flap was not possible to use due to multiple vein and arterial catheter insertion. After wide debridement, the lower lip defect was reconstructed with the use of a composite dorsalis pedis free flap. The extensor hallucis brevis tendon was harvested with the flap and used to support the lower lip in the same manner as the palmaris longus tendon on the radial forearm flap (Figures 4 and 5). Microanastomoses were performed between the dorsalis pedis and facial artery and vein. A second vein anastomosis between the greater saphenous vein, which was harvested with the flap, and the internal jugular vein of the contralateral side was made, in order to reassure good venous drainage and therefore high flap viability. The mental nerve was anastomosed to a branch of the deep peroneal nerve. The patient suffered no complications during post-op period, the flap survived, and the functional result was satisfactory (Figure 6). No foot disability was observed.

## 3. Discussion 

Reconstruction of large lip defects after tumor excision continues to be a great challenge for the otolaryngologist and the plastic surgeon, in order to achieve an acceptable cosmetic and functional result. Restoration of oral lining, external skin, competence, and dynamic function (i.e., articulation, speech, and mastication) are the primary goals in order to avoid post-op complications such as microstomia, drooling, and poor cosmesis. If the defect accounts for more than 80% of the lip, distant free flaps should be used for reconstruction. Local flaps are not effective and the use of regional flaps (deltopectoral, pectoralis major) includes a two-staged procedure and has poor cosmetic and functional results. Since its advent first by Sakai et al. [6] and Sadove et al. [7], the composite radial forearm-longus palmaris free flap has proved to be very versatile and therefore popular in lip reconstruction [4–9]. In short, the folded radial forearm skin serves the cheek and mucosal resurfacing and the ends of the vascularized longus palmaris tendon are anchored either to the cheek or modiolus to support the lip. The technique has undergone several refinements by various authors resulting in multiple variations. Those variations include attaching the tendon to the nasolabial area [6], bilateral modiolus [7], malar periosteum [10], philtral columns [5], and the transferred masseter muscle [11]. Although true dynamic reconstruction can only be achieved with much more complicated techniques [12], the above modifications have resulted in a satisfactory dynamic function. The use of the radial forearm flap has been established in our institution as the basic strategy for the reconstruction of large defects after the removal of extended lower lip carcinomas.

It is significant to mention though the proper donor site vein selection. The vein comitante of the radial artery is not always of reliable size and it is important to plan the flap in order to include large subcutaneous veins or even cephalic vein. This was the cause of failure in our case.

It is also important in the initial planning not to destroy possible options as the opposite donor site. This was the reason in our case to choose the second in order reconstructive option and not to go for the contralateral radial forearm flap.

The composite dorsalis pedis free flap, introduced by Ohmori and Harii [13], can be used in the same manner as radial forearm free flap. These two flaps have a lot in common. They both include pliable and thin skin that can be folded to reconstruct full thickness lower lip defects, good colour match, reliable vascularity, sensitivity, and also tendon structure to support the flap, characteristics that are desirable for a free flap in reconstruction [14]. The dorsalis pedis flap has been used in cases of lip reconstruction but these reports are scarce [15, 16].

The major disadvantage of dorsalis pedis flap comparing to radial forearm is possible donor site morbidity, including scarring, pain, delayed healing, and walking difficulties [17]. It remains though a useful alternative when radial forearm flap fails or it cannot be used such as in the case of a positive Allen test. In our case, the patient did not complain of drooling and did not mention problematic mastication and speech, oral competence, and walking problems.

The anterolateral thigh flap can also be used but the skin paddle is thick and a lot of defatting procedures may be needed [18]. Thus, we consider it as a third choice after the radial forearm and the dorsalis pedis flap.

In conclusion, the dorsalis pedis flap can be used successfully as a second line option for the reconstruction of the lower lip, due to high donor site morbidity. The free radial forearm flap remains the golden standard. Combined approach by an otolaryngologist and a plastic surgeon is also mandatory.
